# Metabolic Hostile Takeover: How Influenza Virus Reprograms Cellular Metabolism for Replication

**DOI:** 10.3390/v17101386

**Published:** 2025-10-17

**Authors:** Xianfeng Hui, Xiaowei Tian, Shihuan Ding, Ge Gao, Xin Zhao, Jiyan Cui, Yiru Hou, Tiesuo Zhao, Hui Wang

**Affiliations:** 1 Department of Immunology, School of Basic Medical Sciences, Henan Medical University, Xinxiang 453003, China; 2 Xinxiang Engineering Technology Research Center of Immune Checkpoint Drug for Liver-Intestinal Tumors, Henan Medical University, Xinxiang 453003, China; 3 Department of Pathogenic Biology, School of Basic Medical Sciences, Henan Medical University, Xinxiang 453003, China; 4 Henan Collaborative Innovation Center of Molecular Diagnosis and Laboratory Medicine, School of Medical Technology, Henan Medical University, Xinxiang 453003, China

**Keywords:** influenza virus, cellular metabolism, glycolysis, lipid biosynthesis, amino acid metabolism, antiviral strategies

## Abstract

Influenza viruses are adept at hijacking host cellular machinery to facilitate their replication and propagation. A critical aspect of this hijacking involves the reprogramming of host cell metabolism. This review summarizes current findings on how influenza virus infection alters major metabolic pathways, including enhanced glycolysis, suppression of oxidative phosphorylation, diversion of TCA cycle intermediates for biosynthesis, and upregulation of lipid and amino acid metabolism. Key nutrients like glucose, glutamine, and serine are redirected to support viral RNA synthesis, protein production, and membrane formation. Moreover, these metabolic changes also modulate host immune responses, potentially aiding in immune evasion. We highlight the role of transcription factors such as SREBPs in lipid synthesis and the impact of one-carbon metabolism on epigenetic regulation. Finally, we discuss how targeting virus-induced metabolic shifts, using agents like 2-deoxyglucose or fatty acid synthesis inhibitors, offers promising avenues for antiviral intervention, while emphasizing the need for selective approaches to minimize harm to normal cells.

## 1. Introduction

Influenza virus, as a major pathogen that has long posed a threat to global public health, causes approximately 1 billion seasonal infections annually, with severe cases reaching 3 to 5 million and related deaths ranging from 290,000 to 650,000 [1,2]. Its epidemiological threat is not only reflected in seasonal epidemics but also in its potential to trigger periodic pandemics. The four influenza pandemics since the 20th century, including the 1918 “Spanish flu” which caused approximately 50 million deaths, have fully demonstrated the destructive power of this virus [3]. Despite significant advances in vaccines and antiviral therapies, the continuous evolution of influenza viruses through antigenic drift and antigenic shift presents ongoing challenges for disease prevention and control [4]. Several critical issues remain unresolved in the current research field. Traditional antiviral drug development primarily targets viral components such as neuraminidase and polymerase, which are highly susceptible to mutation-induced resistance. Moreover, existing vaccines provide limited cross-protection due to the extreme variability of viral surface antigens [5]. In addition, the complex interactions between the influenza virus and host factors—including metabolic reprogramming, immune responses, and signaling pathways—are still not fully understood. Therefore, there is an urgent need to explore novel antiviral strategies to counter the persistent threat posed by influenza viruses [6].

In recent years, increasing insights into virus–host interactions have revealed that the successful replication of the influenza virus depends not only on its genomic structure but also on its precise manipulation of host cellular physiology [7,8]. During infection, host cells undergo profound transcriptional, translational, and metabolic reprogramming to meet the demands of viral replication [8,9]. Among these changes, the regulation of host metabolic pathways has emerged as a critical component of the viral life cycle. Upon infecting host cells, influenza viruses profoundly alter intracellular metabolic states—a phenomenon known as metabolic reprogramming [10]. This process involves the viral manipulation of various host metabolic pathways, including glycolysis [10,11], lipid biosynthesis [12], amino acid metabolism [13,14], and mitochondrial function [15]. Through this reprogramming, the virus secures the energy, biosynthetic precursors, and cellular infrastructure necessary for its replication, assembly, and release.

For instance, influenza virus infection can markedly upregulate glycolysis in host cells. By enhancing glycolytic flux, the virus not only boosts ATP production efficiency but also supplies essential carbon skeletons and energy reserves necessary for viral RNA synthesis and protein translation [16]. Simultaneously, the virus finely tunes lipid metabolic networks to promote the biosynthesis of cholesterol and fatty acids for the construction of the viral envelope, while also preserving the structural stability of the viral replication complex [17]. Moreover, the virus reprograms amino acid metabolism—particularly glutaminolysis and branched-chain amino acid catabolism—to ensure a sufficient substrate supply for the rapid synthesis of viral proteins [18]. Importantly, this metabolic hijacking is not a simple global activation of host metabolism but exhibits pronounced spatiotemporal specificity and dynamic regulation. Glycolysis is preferentially activated during the early stages of infection, lipid biosynthesis is enhanced at intermediate stages, and amino acid utilization becomes predominant in the late phase—precisely aligning with the sequential demands of the viral replication cycle. Moreover, metabolic pathways serve not only as a “resource pool” for viral replication but also as strategic platforms for evading host immune recognition [7,9].

Furthermore, accumulating evidence demonstrates that influenza virus infection induces profound and coordinated metabolic reprogramming across multiple biological levels. These alterations are not limited to immortalized or specific cell lines but are consistently observed in diverse primary cell types, including airway epithelial cells, macrophages, and dendritic cells, indicating a conserved host metabolic response to viral invasion [19]. Beyond the cellular level, systemic metabolic disturbances—such as dysregulated lipid, glucose, and amino acid metabolism—have been reported in both infected animal models and human patients [20]. These systemic alterations likely represent the integrated consequences of viral replication demands, immune activation, and host homeostatic adaptation, collectively highlighting the pivotal role of host metabolism in determining the progression and outcome of influenza virus infection.

Correspondingly, recent studies have demonstrated that disrupting key metabolic enzymes or pathways can effectively limit influenza virus replication and enhance host immune responses, thereby offering novel avenues for the development of antiviral therapies [6,9]. In summary, the influenza virus exploits metabolic reprogramming to achieve a “metabolic hostile takeover” of host cells, a strategy that plays a crucial role throughout various stages of the viral replication cycle. A systematic understanding of virus-induced metabolic reprogramming will facilitate the identification of novel antiviral targets from a metabolic perspective, offering promising avenues for the development of broad-spectrum antiviral drugs and the enhancement of vaccine efficacy. This review aims to provide a comprehensive overview of the major metabolic alterations during influenza virus infection, the associated signaling mechanisms, their impact on viral replication and host immunity, and the potential implications for antiviral therapeutic strategies.

## 2. Glycolysis and Glucose Metabolic Reprogramming Following Influenza Virus Infection

Glycolysis is one of the primary pathways by which cells generate energy, converting glucose into pyruvate with concomitant production of ATP and metabolic intermediates [21]. Under normal aerobic conditions, most cells preferentially channel pyruvate into the mitochondrial tricarboxylic acid (TCA) cycle for complete oxidation and maximal ATP yield [21,22]. However, under certain pathological states or specialized demands, cells may rely predominantly on glycolysis despite the presence of oxygen—a phenomenon known as aerobic glycolysis or the Warburg effect [23]. Recent studies have demonstrated that this metabolic shift is not unique to cancer cells but also occurs broadly in virus-infected host cells, notably during influenza virus infection [10,24].

The influenza virus is a rapidly replicating pathogen with high demands for energy and biosynthetic precursors. To support its life cycle, the virus actively reprograms host cell metabolism [9,25]. One of the major metabolic pathways altered upon infection is glycolysis. During the early stages of infection, the virus enhances the host cell’s capacity for glucose uptake, primarily by upregulating the expression of glucose transporters, particularly GLUT1 and GLUT3. This mechanism enables the host cell to rapidly absorb exogenous glucose, thereby providing sufficient substrates for subsequent glycolytic processes. In addition, the influenza virus enhances the expression and activity of key glycolytic enzymes, including hexokinase 2 (HK2), phosphofructokinase (PFK), pyruvate kinase M2 (PKM2), and lactate dehydrogenase A (LDHA) [10,26,27]. The upregulation of these enzymes facilitates the rapid conversion of glucose to pyruvate, which is subsequently metabolized to lactate rather than entering the mitochondria for complete oxidation—an effect characteristic of the Warburg phenotype. Although this shift sacrifices the efficiency of ATP generation per molecule of glucose, it markedly increases the rate of energy production and supplies a wide range of metabolic intermediates, such as ribose phosphates, lipid precursors, and amino acid substrates, all of which are essential for the synthesis of viral RNA, proteins, and envelope components. Furthermore, lactate accumulation can modulate intracellular pH and the metabolic milieu, indirectly interfering with host immune signaling pathways—for instance, by suppressing the activation of type I interferon responses—thereby enhancing viral replication [28,29].

**Figure 1:**
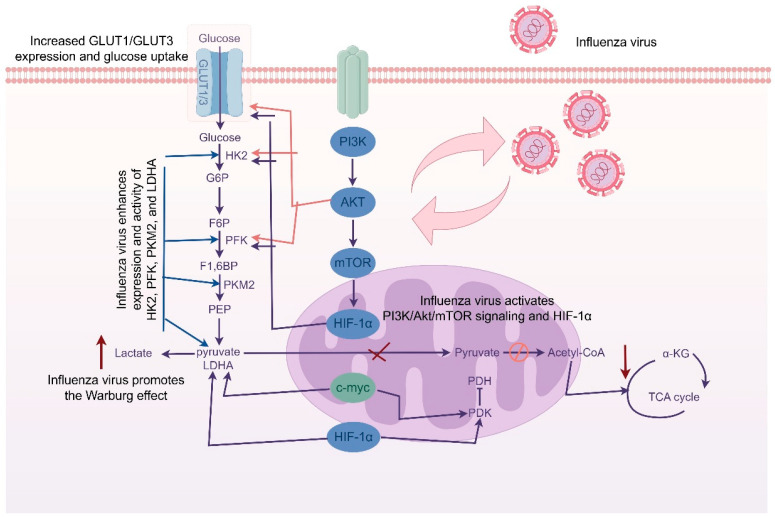
Glucose Metabolic Reprogramming in Influenza Virus Infection.

At the mechanistic level, studies have shown that influenza virus infection activates multiple signaling pathways to regulate the expression of glycolysis-related genes. Among these, the PI3K/Akt/mTOR signaling axis and the HIF-1α pathway are widely recognized as key regulators of glycolytic enhancement [11,26]. The Akt pathway promotes the expression of glucose transporters (GLUTs) and glycolytic enzymes such as HK2, while HIF-1α, a transcription factor, can directly induce the expression of multiple glycolytic genes—even under normoxic conditions—through virus-mediated activation [30,31,32] (Figure 1). In addition, emerging evidence suggests that the influenza virus non-structural protein NS1 may directly modulate the expression of host metabolic genes, further supporting the notion that the virus plays an active role in metabolic reprogramming. For example, studies have shown that NS1 can interact with components of the PI3K/Akt/mTOR signaling pathway. PI3Ks are highly regulated lipid kinases that serve as critical nodes within multiple cellular signaling networks, thereby enhancing glycolytic flux and lipid biosynthesis to meet the metabolic demands of viral replication. These findings highlight NS1 as a multifunctional regulator that tightly links viral replication with host metabolic reprogramming [33,34,35]. However, there is currently no systematic evidence demonstrating coordinated interactions among viral proteins in hijacking host metabolic pathways.

Moreover, pharmacological or genetic interference with the glycolytic pathway has been shown to markedly suppress influenza virus replication. For instance, treatment with the glycolysis inhibitor 2-deoxy-D-glucose (2-DG) significantly lowers viral titers and enhances host immune responses [36,37]. These findings indicate that glycolysis is not only essential for viral replication but may also represent a promising therapeutic target for future anti-influenza interventions.

In summary, the influenza virus rewires host glucose metabolism by upregulating glucose uptake, activating glycolytic enzymes, and inducing aerobic glycolysis (the Warburg effect), thereby supplying the energy and biosynthetic precursors required for efficient viral replication. This metabolic remodeling not only facilitates completion of the viral life cycle but may also modulate host antiviral immune responses. Elucidating the molecular underpinnings of this process will aid in the development of metabolism-targeted anti-influenza strategies—an especially critical need given the rapid evolution of influenza viruses and the diminishing efficacy of conventional antivirals.

## 3. Influenza Virus Infection and Its Crosstalk with the TCA Cycle and Mitochondrial Function

**Figure 2:**
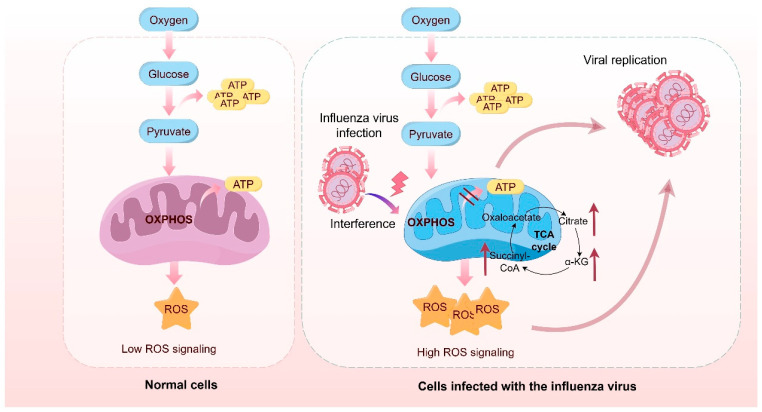
Schematic Representation of TCA Cycle and Mitochondrial Dysregulation During Influenza Virus Infection.

Influenza virus infection not only elicits a robust host immune response but also profoundly remodels mitochondrial function and the regulation of the TCA cycle in host cells [14,38,39]. As the central hub of cellular energy metabolism, mitochondria play critical roles in cell survival, apoptosis, and the orchestration of antiviral immune responses [40,41]. Recent studies have revealed that the influenza virus interferes with mitochondrial oxidative phosphorylation (OXPHOS) through multiple mechanisms, while simultaneously enhancing the anabolic branches of the TCA cycle, thereby reprogramming the host metabolic environment to favor viral replication and immune evasion [14] (Figure 2).

Under normal physiological conditions, the TCA cycle operates in the presence of oxygen, generating NADH and FADH_2_, which fuel ATP synthesis via the mitochondrial electron transport chain (ETC) [42]. This process constitutes the primary source of cellular energy. However, during influenza virus infection, OXPHOS efficiency is generally impaired. This inhibition is believed to stem, at least in part, from the direct disruption of mitochondrial structure and function by viral proteins such as PB1-F2 and NS1 [26,43]. PB1-F2, for example, localizes to mitochondria and induces a loss of mitochondrial membrane potential, disrupts cristae architecture, and suppresses ETC activity, ultimately impairing ATP production [44,45,46]. Although this reduction in bioenergetic capacity is detrimental to host cells, it may benefit the virus by attenuating the host’s ability to synthesize antiviral proteins and by establishing a metabolic state that favors viral replication [47].

Despite compromised OXPHOS, levels of certain TCA cycle intermediates—such as citrate, α-ketoglutarate, and succinate—are often elevated during infection. These metabolites play diverse roles in supporting viral replication [39,47]. Citrate can be exported to the cytosol and utilized for de novo fatty acid and cholesterol synthesis, contributing to the formation of viral envelope components [48,49,50]. α-Ketoglutarate and succinate are also implicated in modulating redox balance and epigenetic regulation, including histone demethylation [51]. Moreover, influenza infection enhances anabolic offshoots of the TCA cycle, such as glutaminolysis and the serine–one-carbon metabolism pathway, which supply carbon substrates and reducing equivalents (e.g., NADPH) essential for biosynthesis and antioxidant defense during viral replication [49,52].

Mitochondrial dysfunction induced by the influenza virus further disrupts cellular redox homeostasis. Infection often leads to elevated levels of mitochondrial reactive oxygen species (mtROS), disturbing redox equilibrium [53,54,55]. While moderate ROS levels can activate antiviral responses through pathways like NF-κB and type I interferon signaling, excessive ROS contributes to oxidative stress, apoptosis, and even cytokine storm development, thereby exacerbating tissue injury [56]. Notably, the virus may exploit ROS modulation to evade immune detection. The NS1 protein, for instance, suppresses the RIG-I/MAVS signaling axis—an antiviral pathway localized on the mitochondrial outer membrane that is ROS-dependent—thereby delaying type I interferon production and dampening early immune responses [57,58].

Additionally, mitochondria play a key role in the regulation of programmed cell death, particularly intrinsic apoptosis [59]. Influenza virus can manipulate this process by altering the expression of Bcl-2 family proteins, increasing mitochondrial outer membrane permeability, and promoting cytochrome c release [60,61,62]. While virus-induced apoptosis may facilitate progeny virion release, excessive cell death could also trigger strong inflammatory responses [63]. Hence, the virus appears to fine-tune apoptotic signaling to balance cell death with the preservation of a favorable replication environment.

In conclusion, the influenza virus employs a multifaceted strategy to manipulate host mitochondrial function and TCA cycle activity, thereby reshaping cellular metabolism in a manner that supports viral replication while subverting host immune defenses. This regulation extends beyond energy metabolism to encompass redox control, immune modulation, and apoptotic pathways, highlighting the central role of mitochondria in the pathogenesis of influenza infection.

## 4. The Interplay Between Influenza Virus Infection and Lipid Metabolism

Lipid metabolism is fundamental for maintaining normal cellular structure and function and plays a critical role in the life cycle of viruses [64], particularly enveloped viruses such as the influenza virus [65,66]. The influenza virus relies heavily on host lipid metabolism for multiple stages of its infection cycle, including viral entry, replication, assembly, and budding. Increasing evidence has shown that the influenza virus actively reprograms host lipid metabolic pathways upon infection, notably through the activation of the sterol regulatory element-binding proteins (SREBPs) signaling pathway, which enhances fatty acid and cholesterol biosynthesis [65]. This metabolic remodeling creates a lipid-rich intracellular environment that supports efficient viral replication and propagation. Understanding how the influenza virus hijacks these lipid metabolic processes is essential for uncovering its pathogenic strategies and identifying novel therapeutic targets.

Lipids play multifaceted and indispensable roles throughout the entire influenza virus life cycle. The viral envelope is derived from the host cell plasma membrane, which is enriched in phospholipids, cholesterol, and sphingolipids—key components that ensure the structural integrity and infectivity of virions [67]. During replication and assembly, lipids contribute to the formation and remodeling of virus-associated membranous compartments, including the endoplasmic reticulum (ER), the ER-Golgi intermediate compartment (ERGIC), and lipid droplets. These membrane-bound organelles serve as critical sites for viral RNA replication, protein synthesis, and virion morphogenesis [68,69]. Moreover, the specific lipid composition of host membranes, particularly the presence of cholesterol-rich lipid rafts, directly affects the efficiency of viral budding and the functional properties of progeny virions [70,71]. Cholesterol is especially vital for regulating membrane fluidity and maintaining lipid raft structure, which facilitates the spatial organization required for optimal virion assembly and release [72].

**Figure 3:**
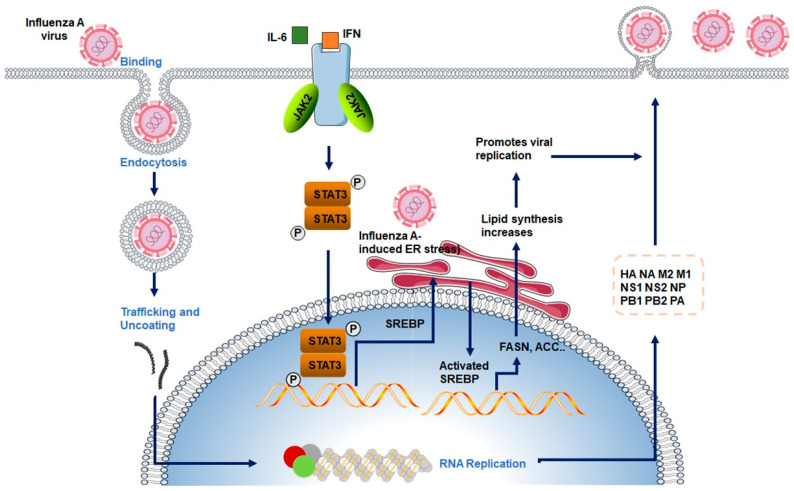
Influenza Virus–Induced Activation of SREBPs and Upregulation of Lipogenic Pathways.

To promote lipid biosynthesis, the influenza virus activates SREBPs, a family of membrane-bound transcription factors that regulate lipid metabolism [73]. Among the SREBP isoforms, SREBP-1c is primarily involved in fatty acid biosynthesis, whereas SREBP-2 regulates cholesterol synthesis via target genes such as 3-hydroxy-3-methylglutaryl-CoA reductase (HMGCR) [74]. Under homeostatic conditions, SREBPs are retained in an inactive form in the ER through interaction with SREBP cleavage-activating protein (SCAP), and their activation is tightly controlled by intracellular cholesterol levels [74,75]. Upon influenza virus infection, several host signaling cascades—such as ER stress induction, mechanistic target of rapamycin (mTOR) pathway activation, and AMP-activated protein kinase (AMPK) inhibition—are triggered to promote SREBP cleavage, nuclear translocation, and transcriptional activation of lipogenic genes, including FASN and acetyl-CoA carboxylase (ACC) [7,73]. As a result, cellular lipid synthesis is significantly upregulated (Figure 3).

Experimental evidence supports the sustained activation of the SREBP pathway during influenza infection. For example, infection with the H1N1 virus has been shown to increase the expression of key lipogenic enzymes such as FASN, ACC, and stearoyl-CoA desaturase 1 (SCD1) (Cited from Keystone Symposia). The viral non-structural protein NS1 may also facilitate SREBP activation by modulating host signaling pathways [65,76]. Additionally, cytokines released during the inflammatory response—such as IL-6—can upregulate SREBP-1c expression via the JAK/STAT3 axis, further amplifying lipid biosynthesis and accumulation in infected cells [77,78].

Both fatty acid and cholesterol synthesis play crucial roles in supporting influenza virus replication. Fatty acids are essential not only as building blocks of membranes but also as precursors for complex lipids such as phospholipids, sphingolipids, and triglycerides, which contribute to membrane curvature, fusion, and intracellular signaling [79,80]. Viral infection drives de novo fatty acid synthesis to support membrane expansion and remodeling required for viral replication complex formation [81]. Pharmacological inhibition of FASN using agents like C75 or Orlistat significantly impairs viral replication, highlighting the functional importance of fatty acid metabolism in the influenza life cycle [65]. Similarly, cholesterol biosynthesis is tightly linked to viral budding and infectivity. As a key component of lipid rafts, cholesterol maintains the structural integrity of budding sites and influences virion release. Cholesterol- and sphingolipid-enriched lipid rafts serve as preferred platforms (“budozones”) for the concentration of viral glycoproteins (HA/NA) and matrix proteins during virion assembly. Disruption of raft integrity through cholesterol depletion alters glycoprotein sorting, membrane order, and envelope stability, thereby affecting both particle release and infectivity [82]. Notably, the effect of membrane cholesterol on budding efficiency is non-linear and context-dependent: modest or short-term depletion (e.g., low concentrations or brief exposure to methyl-β-cyclodextrin, MβCD) may increase particle release, but these virions are often structurally defective and less infectious [83]; in contrast, severe depletion or prolonged treatment impairs proper assembly and reduces infectious yield. Conversely, exogenous cholesterol can restore membrane order and virion integrity but may also suppress particle release in some contexts, suggesting that excessively high membrane rigidity can hinder late stages such as membrane scission or virion detachment [72].

Inhibitors of cholesterol synthesis, such as statins (e.g., simvastatin, lovastatin), have shown in vitro antiviral effects, presumably by depleting membrane cholesterol and disrupting the budding process [84]. Although further clinical studies are needed, these findings underscore the therapeutic potential of targeting lipid metabolism, particularly fatty acid and cholesterol synthesis, in antiviral strategies against influenza.

Beyond biosynthetic pathways, lipid metabolism also supports the formation of intracellular organelles required for viral replication [69,85]. Lipid droplets (LDs), which are rich in triglycerides and cholesteryl esters, are important for energy homeostasis and immune regulation [86,87]. Upon influenza virus infection, host cells accumulate LDs, which may serve as lipid reservoirs, anchoring sites for replication complexes, or modulators of antiviral responses [63,88]. In parallel, the virus-induced activation of lipid metabolism contributes to ER expansion and Golgi dilation, remodeling the host cell’s membrane infrastructure to create favorable conditions for viral RNA replication and protein trafficking [85,89]. These alterations reflect a comprehensive reprogramming of host cellular architecture to support efficient viral reproduction.

Given the central role of lipid metabolism in influenza virus infection, targeting lipid metabolic pathways has emerged as a promising antiviral strategy. Several drugs have become candidate inhibitors of influenza virus replication by interfering with key metabolic nodes. Inhibitors of fatty acid synthesis (e.g., Orlistat), cholesterol production (e.g., Statins), SREBP activation (e.g., Betulin), and AMPK modulation (e.g., Metformin) have demonstrated antiviral activity by disrupting critical lipid-dependent steps in the viral life cycle [65,90]. As summarized in Table 1, these agents reduce viral replication in both in vitro and in vivo models, supporting the rationale for further exploration of host-directed lipid metabolism inhibitors as broad-spectrum therapeutics against influenza and potentially other enveloped viruses.

## 5. Interplay Between Influenza Virus Infection and Amino Acid Metabolism and One-Carbon Metabolism

Amino acid metabolism and its interconnected one-carbon metabolism are essential for cellular homeostasis, supporting biosynthesis, epigenetic regulation, and redox balance [93]. Recent studies have uncovered that influenza virus infection extensively reprograms host amino acid metabolic networks, particularly those involving glutamine and serine metabolism [7,9]. This rewiring not only meets the increased demand for nucleotides, proteins, and energy required during rapid viral replication but also shapes the host epigenetic landscape via one-carbon metabolism, thereby contributing to immune modulation and evasion.

Amino acids serve as fundamental biosynthetic precursors during viral replication. In addition to supporting viral protein synthesis, specific amino acids contribute to anaplerotic input into the TCA cycle, generate reducing equivalents such as NADPH and glutathione, and fuel nucleotide biosynthesis [9]. Influenza virus infection has been shown to upregulate host amino acid transporters, including SLC1A5 and SLC7A5, thereby enhancing the cellular uptake of glutamine, serine, and other amino acids essential for viral propagation [12]. Among these, glutamine and serine act as central metabolic hubs, linking biosynthetic and antioxidant pathways critical for sustaining infection.

**Figure 4:**
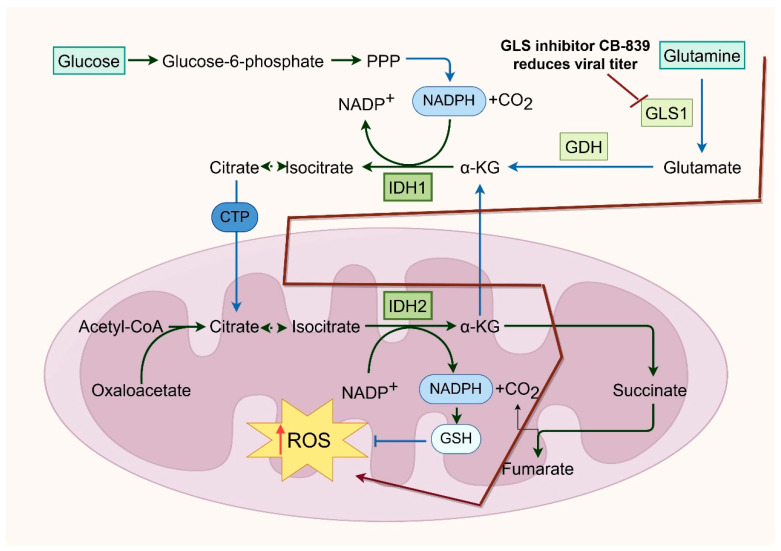
Glutamine Metabolism Supports Influenza Virus Replication.

Glutamine, the most abundant non-essential amino acid in mammalian cells, plays a crucial role in protein synthesis, energy production, and nitrogen metabolism [94,95]. During influenza virus infection, glutamine metabolism is significantly upregulated. Glutaminase (GLS) converts glutamine into glutamate, which can be further metabolized into α-ketoglutarate to fuel the TCA cycle, thus supporting energy generation and redox homeostasis [6,9]. Glutamine also donates nitrogen for de novo purine and pyrimidine biosynthesis—processes essential for viral RNA synthesis. In parallel, glutamate contributes to the synthesis of glutathione, a major intracellular antioxidant that helps counteract virus-induced oxidative stress [96,97,98]. Pharmacological inhibition of glutaminolysis, such as with GLS inhibitors like CB-839, significantly reduces influenza viral titers, emphasizing the indispensable role of glutamine metabolism in viral replication [12]. Additionally, viral infection may promote glutamine catabolism by inducing host transcription factors such as c-Myc or modulating GLS expression to create a metabolically favorable environment for replication [9,99] (Figure 4).

Similarly, serine metabolism is upregulated in various infections to support nucleotide biosynthesis and maintain redox homeostasis [27]. Serine is synthesized from the glycolytic intermediate 3-phosphoglycerate through a three-step enzymatic pathway involving phosphoglycerate dehydrogenase (PHGDH), phosphoserine aminotransferase (PSAT1), and phosphoserine phosphatase (PSPH) [100,101]. Serine is subsequently converted into glycine by serine hydroxymethyltransferase (SHMT), donating one-carbon units into the tetrahydrofolate (THF) cycle. These one-carbon units, particularly 5,10-methylene-THF, are essential for purine and thymidine synthesis and thus critical for viral genome replication. Furthermore, serine-derived NADPH production aids in the maintenance of redox homeostasis under infection-induced oxidative stress.

One-carbon metabolism not only fuels nucleotide biosynthesis but also supports the generation of S-adenosylmethionine (SAM), the universal methyl donor involved in DNA, RNA, and histone methylation [102]. These epigenetic modifications influence gene expression, chromatin organization, and immune regulation. Influenza virus infection increases one-carbon flux, enhancing SAM availability and potentially remodeling the host epigenome. For example, upregulated DNA and histone methyltransferase activity during infection can lead to hypermethylation of promoter regions of interferon-stimulated genes (ISGs), silencing antiviral responses and facilitating immune evasion [103,104]. Viral proteins such as NS1 have been shown to interact with host epigenetic regulators and perturb SAM metabolism, potentially altering miRNA processing and RNA methylation patterns, further expanding the virus’s ability to manipulate host gene expression through one-carbon metabolic rewiring [105].

The crosstalk between amino acid and one-carbon metabolism also has profound immunological implications. Glutamine is essential for the activation and function of immune cells such as T lymphocytes and macrophages, influencing their proliferation, migration, and cytokine production [106,107]. The serine–one-carbon axis supports DNA synthesis and cell cycle progression in proliferating immune cells, including effector T cells [108]. Influenza-induced disruption of these pathways can lead to immune dysfunction. For instance, glutamine depletion diminishes CD8^+^ T cell effector responses, while enhanced one-carbon metabolism may favor regulatory T cell (Treg) differentiation, suppress inflammation, and contribute to viral immune escape [109,110]. Through these multifaceted mechanisms, the influenza virus not only exploits host amino acid metabolism for replication but also dampens antiviral immunity by reshaping metabolic and epigenetic landscapes.

## 6. Interplay Between Metabolic Reprogramming and Host Immune Responses During Influenza Virus Infection

**Figure 5:**
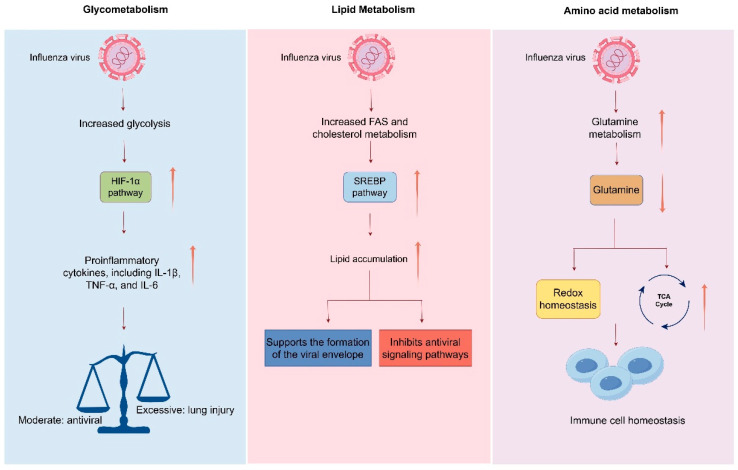
Influenza-Induced Metabolic Reprogramming and Immune Dynamics.

During viral infection, the reprogramming of host cell metabolism not only supplies the energy and biosynthetic precursors necessary for viral replication but also profoundly influences the host immune response. Influenza virus infection rapidly reprograms host cell metabolism, establishing a new equilibrium between metabolic processes and immune responses. For instance, enhanced glycolysis activates hypoxia-inducible factor 1-alpha (HIF-1α), which upregulates the expression of proinflammatory cytokines such as IL-1β, TNF-α, and IL-6 [111]. Meanwhile, pathways involved in fatty acid synthesis (FAS) and cholesterol metabolism play dual roles in regulating both the interferon response and inflammatory balance [112]. The virus can activate the sterol regulatory element-binding protein (SREBP) pathway to promote lipid accumulation, which not only supports the formation of the viral envelope but also suppresses antiviral signaling cascades mediated by Toll-like receptors (TLRs) and retinoic acid-inducible gene I (RIG-I), thereby facilitating immune evasion. Amino acid metabolism—particularly that of glutamine—also contributes to the regulation of immune responses. Glutamine metabolism sustains immune cell activity by supporting redox homeostasis and fueling the TCA cycle, while one-carbon units derived from serine are critical for the epigenetic regulation (e.g., DNA methylation) required for T cell differentiation [113,114]. Influenza virus may manipulate these metabolic pathways to alter immune cell lineage commitment and functional states (Figure 5).

By reshaping host metabolism, the influenza virus not only meets its own replicative needs but may also indirectly suppress immune detection and clearance. For example, virus-induced mitochondrial dysfunction has been shown to impair the formation of mitochondrial antiviral signaling (MAVS) complexes, thereby reducing type I interferon production. Additionally, as previously mentioned, lactate accumulation can suppress the activation of NF-κB, thereby downregulating the transcription of antiviral genes. Metabolic perturbations may also disrupt intercellular immune crosstalk—for instance, by inhibiting antigen presentation by dendritic cells or suppressing the activity of natural killer (NK) cells—thereby promoting viral persistence within the host [115].

## 7. Therapeutic Implications: Targeting Host Metabolism as an Antiviral Strategy Against Influenza

Conventional influenza therapies primarily rely on antiviral agents that directly target viral components, such as neuraminidase inhibitors (e.g., oseltamivir) and polymerase inhibitors (e.g., baloxavir marboxil) [116,117,118]. However, the high mutation rate and genetic variability of influenza viruses pose significant challenges for these direct-acting antivirals, particularly during widespread outbreaks or infections with highly pathogenic avian influenza strains [119]. Consequently, increasing attention has turned toward host-directed therapies, with a particular focus on metabolic pathways, offering novel avenues and targets for antiviral intervention.

Influenza virus infection induces extensive reprogramming of host cellular metabolism, markedly activating glycolysis, lipid biosynthesis, and amino acid metabolism to meet the biosynthetic and energetic demands of viral RNA replication, protein synthesis, and envelope formation. Since viruses are disproportionately dependent on these metabolic activities compared to quiescent host cells, selectively targeting key metabolic enzymes or pathways holds promise for impairing viral replication without severely disrupting host cellular function.

In addition, interventions targeting glutamine metabolism, serine biosynthesis, and the one-carbon (1C) metabolic network are being explored for metabolic modulation-based antiviral therapy. For instance, inhibition of serine biosynthesis impairs nucleotide production and redox homeostasis, thereby creating a less favorable environment for viral replication [24,120]. These metabolic strategies may also modulate host immune responses, offering potential benefits in reducing immunopathology associated with severe influenza infections.

Despite encouraging results in vitro and in animal models, translating host-targeted metabolic therapies into clinical practice presents several challenges, particularly with regard to specificity. On one hand, metabolism is fundamental to cellular physiology, and broad inhibition of metabolic pathways may result in toxicity, especially in rapidly proliferating tissues such as the gut epithelium or hematopoietic system [121]. On the other hand, viral dependency on host metabolism is cell type- and infection stage-specific, highlighting the need to identify and target “metabolic vulnerabilities” that are selectively required for viral replication [49].

## 8. Conclusions and Future Perspectives

Influenza viruses systematically reprogram the host’s metabolic network to meet their needs for replication, survival, and propagation—a process that can be metaphorically termed “metabolic hostile takeover.” From enhancing glycolysis and suppressing oxidative phosphorylation to promoting anabolic pathways for lipids, amino acids, and nucleotides, influenza viruses skillfully manipulate the host cell’s resource supply system, effectively transforming it into a “factory serving viral replication.” This metabolic reprogramming not only provides the material foundation for viral replication but may also modulate host immune responses and cell fate, thereby further enhancing viral persistence and pathogenicity.

Current research has unveiled critical alterations in various metabolic pathways during influenza virus infection, including glycolysis, the TCA cycle, lipid biosynthesis, one-carbon metabolism, and glutamine metabolism. By hijacking these pathways, influenza viruses reshape the host cell’s energy metabolism, redox balance, epigenetic modifications, and signaling networks. These changes are not isolated events but are tightly coupled with different stages of the viral replication cycle, even contributing to immune evasion. Thus, metabolic remodeling is not merely a “byproduct” of infection but rather a central battlefield in virus–host interactions.

While many of these metabolic alterations are transient and reversible under mild or acute infection conditions, severe or high-burden infections can induce sustained metabolic dysregulation. Such persistent disturbances are primarily driven by ongoing viral replication, mitochondrial impairment, and prolonged inflammatory signaling, which collectively disrupt cellular homeostasis and may lead to irreversible tissue damage. These observations suggest that the extent and duration of metabolic reprogramming are largely determined by viral load and host resilience, thereby explaining why the metabolic consequences of infection can span a spectrum from adaptive to pathogenic outcomes.

Recent studies have demonstrated that metabolic reprogramming is a common feature of host responses to various viral infections, including the influenza virus, SARS-CoV-2, respiratory syncytial virus (RSV), and dengue virus (DENV). These viruses induce similar shifts toward enhanced glycolysis, lipid biosynthesis, and amino acid metabolism to meet the bioenergetic and biosynthetic demands of viral replication. For instance, SARS-CoV-2 infection has been shown to significantly alter lipid patterns in plasma, correlating with disease severity [122]. Similarly, RSV infection drives metabolic reprogramming in pediatric airways, supporting a glycolytic phenotype [123]. DENV infection imposes significant metabolic changes, including glycolytic upregulation and lipid droplet utilization through lipophagy [124]. However, influenza viruses also display unique metabolic signatures that distinguish them from other respiratory viruses. For instance, influenza infection markedly perturbs mitochondrial dynamics and oxidative phosphorylation, alters fatty acid oxidation, and reorganizes cholesterol-rich lipid rafts to facilitate viral assembly and budding. These distinct metabolic modulations reflect the virus’s specific replication strategy and its interaction with host immune signaling. Understanding the balance between shared and virus-specific metabolic pathways will be essential for designing broad-spectrum as well as influenza-targeted antiviral strategies.

From a therapeutic standpoint, targeting host metabolic pathways offers a novel strategy beyond conventional antiviral drugs. Unlike direct inhibition of viral proteins, metabolic interventions may reduce the risk of drug resistance caused by viral mutations and could simultaneously achieve viral clearance and tissue protection by modulating the immune microenvironment. To date, several metabolic modulators (e.g., 2-deoxyglucose, fatty acid synthase inhibitors, mTOR inhibitors) have demonstrated antiviral potential in vitro and in animal models. However, significant challenges remain in improving selectivity, minimizing toxicity, and achieving clinical translation.

Here, we boldly propose several key directions that warrant further investigation in future research:

(1) Systematic Mapping of Virus–Host Metabolic Interactions

Integrative multi-omics analyses (metabolomics, transcriptomics, proteomics) will help delineate the spatiotemporal dynamics of metabolic pathways during influenza infection, identifying “metabolic vulnerabilities” that are highly virus-dependent yet tolerable to normal cells. Single-cell technologies will further elucidate cell-type-specific metabolic heterogeneity in response to infection, providing a basis for precision therapeutics.

(2) Functional Validation and Mechanistic Elucidation

While many metabolic changes have been correlated with infection, causal relationships must be established using gene-editing tools (e.g., CRISPR-Cas9), enzyme-specific inhibitors, or metabolic pathway knockout models. Investigating the crosstalk between metabolism and cellular signaling, epigenetics, and organelle function will deepen our understanding of viral exploitation strategies.

(3) Development of Novel Metabolism-Targeting Antivirals

Current metabolic interventions often lack specificity, potentially disrupting normal cellular functions. Future efforts should focus on designing “infection-specific” or “virus-inducible” metabolic modulators. For instance, leveraging virus-induced enzymatic activities or metabolite fluctuations to enable conditional drug activation or inhibition could enhance both antiviral efficacy and safety.

(4) Integration of Metabolic Interventions with Existing Therapies

Metabolic modulators may be combined with conventional antivirals (e.g., neuraminidase inhibitors) for multi-target, multi-pathway suppression. Additionally, they could serve as adjuvants to immunomodulators, mitigating excessive immune responses (e.g., cytokine storms) in late-stage infections, thereby enabling a “dual-track” therapeutic approach that balances antiviral and immunoregulatory effects.

In summary, deciphering how influenza viruses reprogram host metabolism not only advances our understanding of viral pathogenesis and host defense mechanisms but also unveils new therapeutic opportunities. Interdisciplinary research integrating metabolism, immunology, bioinformatics, and drug development will propel the emerging field of “metabolic antivirals” from bench to bedside, ultimately enabling more effective and precise treatments for influenza virus infection.

## Figures and Tables

**Table 1:** Potential Antiviral Agents Targeting Lipid Metabolism.

Target Pathway	Agent	Mechanism of Action	Antiviral Effect	Stage
Fatty acid synthesis	Orlistat	Inhibits FASN, blocks fatty acid biosynthesis	Suppresses viral RNA replication and protein synthesis	Preclinical
Cholesterol synthesis	Statins	Inhibit HMG-CoA reductase, reduce cholesterol synthesis	Impairs viral budding and infectivity	Preclinical/Early clinical
SREBP activation	Betulin	Inhibits SREBP cleavage and nuclear translocation	Decreases lipid synthesis and viral replication	Preclinical
AMPK/mTOR	Metformin	Activates AMPK, inhibits mTOR, suppresses SREBP activity indirectly	Restores metabolic balance, limits viral replication [91]	Early clinical (repurposing) [92]

## References

[1] Lin T.H., Zhu X., Wang S., Zhang D., McBride R., Yu W., Babarinde S., Paulson J.C., Wilson I.A. (2024). A single mutation in bovine influenza H5N1 hemagglutinin switches specificity to human receptors. Science.

[2] Hui X., Cao L., Xu T., Zhao L., Huang K., Zou Z., Ren P., Mao H., Yang Y., Gao S. (2022). PSMD12-Mediated M1 Ubiquitination of Influenza A Virus at K102 Regulates Viral Replication. J. Virol..

[3] He J., Kam Y.W. (2024). Insights from Avian Influenza: A Review of Its Multifaceted Nature and Future Pandemic Preparedness. Viruses.

[4] Iuliano A.D., Roguski K.M., Chang H.H., Muscatello D.J., Palekar R., Tempia S., Cohen C., Gran J.M., Schanzer D., Cowling B.J. (2018). Estimates of global seasonal influenza-associated respiratory mortality: A modelling study. Lancet.

[5] Xu J., Luo Q., Huang Y., Li J., Ye W., Yan R., Zhou X., He Z., Liu G., Zhu Q. (2024). Influenza neuraminidase mutations and resistance to neuraminidase inhibitors. Emerg. Microbes Infect..

[6] Tang H., Jiang F., Zhang Z., Yang J., Li L., Zhang Q. (2025). Metabolism-associated protein network constructing and host-directed anti-influenza drug repurposing. Brief. Bioinform..

[7] Thaker S.K., Ch’ng J., Christofk H.R. (2019). Viral hijacking of cellular metabolism. BMC Biol..

[8] Goodwin C.M., Xu S., Munger J. (2015). Stealing the Keys to the Kitchen: Viral Manipulation of the Host Cell Metabolic Network. Trends Microbiol..

[9] Smallwood H.S., Duan S., Morfouace M., Rezinciuc S., Shulkin B.L., Shelat A., Zink E.E., Milasta S., Bajracharya R., Oluwaseum A.J. (2017). Targeting Metabolic Reprogramming by Influenza Infection for Therapeutic Intervention. Cell Rep..

[10] Ren L., Zhang W., Zhang J., Zhang J., Zhang H., Zhu Y., Meng X., Yi Z., Wang R. (2021). Influenza A Virus (H1N1) Infection Induces Glycolysis to Facilitate Viral Replication. Virol. Sin..

[11] Meng X., Zhu Y., Yang W., Zhang J., Jin W., Tian R., Yang Z., Wang R. (2024). HIF-1alpha promotes virus replication and cytokine storm in H1N1 virus-induced severe pneumonia through cellular metabolic reprogramming. Virol. Sin..

[12] Mayer K.A., Stockl J., Zlabinger G.J., Gualdoni G.A. (2019). Hijacking the Supplies: Metabolism as a Novel Facet of Virus-Host Interaction. Front. Immunol..

[13] Al-Shalan H.A.M., Zhou L., Dong Z., Wang P., Nicholls P.K., Boughton B., Stumbles P.A., Greene W.K., Ma B. (2023). Systemic perturbations in amino acids/amino acid derivatives and tryptophan pathway metabolites associated with murine influenza A virus infection. Virol. J..

[14] Ohno M., Sekiya T., Nomura N., Daito T.J., Shingai M., Kida H. (2020). Influenza virus infection affects insulin signaling, fatty acid-metabolizing enzyme expressions, and the tricarboxylic acid cycle in mice. Sci. Rep..

[15] Pila-Castellanos I., Molino D., McKellar J., Lines L., Da Graca J., Tauziet M., Chanteloup L., Mikaelian I., Meyniel-Schicklin L., Codogno P. (2021). Mitochondrial morphodynamics alteration induced by influenza virus infection as a new antiviral strategy. PLoS Pathog..

[16] Awad K., Abdelhadi M., Awad A.M. (2025). High Glucose Reduces Influenza and Parainfluenza Virus Productivity by Altering Glycolytic Pattern in A549 Cells. Int. J. Mol. Sci..

[17] Zhang J., Wu Y., Wang Y., Liu P., Liu K., Sun J., Zhang P., Wang X., Liu X., Xu X. (2024). Influenza A virus infection activates STAT3 to enhance SREBP2 expression, cholesterol biosynthesis, and virus replication. iScience.

[18] Bezgovsek J., Gulbins E., Friedrich S.K., Lang K.S., Duhan V. (2018). Sphingolipids in early viral replication and innate immune activation. Biol. Chem..

[19] Rezinciuc S., Bezavada L., Bahadoran A., Duan S., Wang R., Lopez-Ferrer D., Finkelstein D., McGargill M.A., Green D.R., Pasa-Tolic L. (2020). Dynamic metabolic reprogramming in dendritic cells: An early response to influenza infection that is essential for effector function. PLoS Pathog..

[20] Ohno M., Gowda S.G.B., Sekiya T., Nomura N., Shingai M., Hui S.P., Kida H. (2023). The elucidation of plasma lipidome profiles during severe influenza in a mouse model. Sci. Rep..

[21] Liu H., Wang S., Wang J., Guo X., Song Y., Fu K., Gao Z., Liu D., He W., Yang L.L. (2025). Energy metabolism in health and diseases. Signal Transduct. Target. Ther..

[22] Lu X., Zhang A., Wang H., Xu X., Chen L., Luo L. (2025). Emerging role of the TCA cycle and its metabolites in lung disease. Front. Physiol..

[23] Mathew M., Nguyen N.T., Bhutia Y.D., Sivaprakasam S., Ganapathy V. (2024). Metabolic Signature of Warburg Effect in Cancer: An Effective and Obligatory Interplay between Nutrient Transporters and Catabolic/Anabolic Pathways to Promote Tumor Growth. Cancers.

[24] Sanchez E.L., Lagunoff M. (2015). Viral activation of cellular metabolism. Virology.

[25] Kim B., Arcos S., Rothamel K., Jian J., Rose K.L., McDonald W.H., Bian Y., Reasoner S., Barrows N.J., Bradrick S. (2020). Discovery of Widespread Host Protein Interactions with the Pre-replicated Genome of CHIKV Using VIR-CLASP. Mol. Cell.

[26] Zhang Y., Chang L., Xin X., Qiao Y., Qiao W., Ping J., Xia J., Su J. (2024). Influenza A virus-induced glycolysis facilitates virus replication by activating ROS/HIF-1alpha pathway. Free Radic. Biol. Med..

[27] Darweesh M., Mohammadi S., Rahmati M., Al-Hamadani M., Al-Harrasi A. (2025). Metabolic reprogramming in viral infections: The interplay of glucose metabolism and immune responses. Front. Immunol..

[28] Thyrsted J., Storgaard J., Blay-Cadanet J., Heinz A., Thielke A.L., Crotta S., de Paoli F., Olagnier D., Wack A., Hiller K. (2021). Influenza A induces lactate formation to inhibit type I IFN in primary human airway epithelium. iScience.

[29] O’Carroll S.M., Henkel F.D.R., O’Neill L.A.J. (2024). Metabolic regulation of type I interferon production. Immunol. Rev..

[30] Agani F., Jiang B.H. (2013). Oxygen-independent regulation of HIF-1: Novel involvement of PI3K/AKT/mTOR pathway in cancer. Curr. Cancer Drug Targets.

[31] Mazurakova A., Koklesova L., Csizmar S.H., Samec M., Brockmueller A., Sudomova M., Biringer K., Kudela E., Pec M., Samuel S.M. (2024). Significance of flavonoids targeting PI3K/Akt/HIF-1alpha signaling pathway in therapy-resistant cancer cells-A potential contribution to the predictive, preventive, and personalized medicine. J. Adv. Res..

[32] Reyes A., Corrales N., Galvez N.M.S., Bueno S.M., Kalergis A.M., Gonzalez P.A. (2020). Contribution of hypoxia inducible factor-1 during viral infections. Virulence.

[33] Ji Z.X., Wang X.Q., Liu X.F. (2021). NS1: A Key Protein in the “Game” Between Influenza A Virus and Host in Innate Immunity. Front. Cell Infect. Microbiol..

[34] Dubois J., Traversier A., Julien T., Padey B., Lina B., Bourdon J.C., Marcel V., Boivin G., Rosa-Calatrava M., Terrier O. (2019). The Nonstructural NS1 Protein of Influenza Viruses Modulates TP53 Splicing through Host Factor CPSF4. J. Virol..

[35] Aslam S., Sanchez-Aparicio M.T., Siempelkamp B.D., Dornan G.L., Tsolakos N., Burke J.E., Hale B.G., Garcia-Sastre A., Ayllon J. (2025). Influenza A virus NS1 protein mimics oncogenic PI3K resulting in isoform specific cellular redistribution and activation. Proc. Natl. Acad. Sci. USA.

[36] Larcombe D.E., Bohovych I.M., Pradhan A., Ma Q., Hickey E., Leaves I., Cameron G., Avelar G.M., de Assis L.J., Childers D.S. (2023). Glucose-enhanced oxidative stress resistance-A protective anticipatory response that enhances the fitness of Candida albicans during systemic infection. PLoS Pathog..

[37] Zevini A., Palermo E., Di Carlo D., Alexandridi M., Rinaldo S., Paone A., Cutruzzola F., Etna M.P., Coccia E.M., Olagnier D. (2022). Inhibition of Glycolysis Impairs Retinoic Acid-Inducible Gene I-Mediated Antiviral Responses in Primary Human Dendritic Cells. Front. Cell Infect. Microbiol..

[38] Huckestein B.R., Alcorn J.F. (2022). Improving Mitochondrial Function in Viral Infection: Targeting Cellular Metabolism. Am. J. Respir. Cell Mol. Biol..

[39] Sanchez-Garcia F.J., Perez-Hernandez C.A., Rodriguez-Murillo M., Moreno-Altamirano M.M.B. (2021). The Role of Tricarboxylic Acid Cycle Metabolites in Viral Infections. Front. Cell Infect. Microbiol..

[40] Changaei M., Azimzadeh Tabrizi Z., Karimi M., Kashfi S.A., Koochaki Chahardeh T., Hashemi S.M., Soudi S. (2025). From powerhouse to modulator: Regulating immune system responses through intracellular mitochondrial transfer. Cell Commun. Signal.

[41] Sun Z., Wang Y., Jin X., Li S., Qiu H.J. (2024). Crosstalk between Dysfunctional Mitochondria and Proinflammatory Responses during Viral Infections. Int. J. Mol. Sci..

[42] Martinez-Reyes I., Chandel N.S. (2020). Mitochondrial TCA cycle metabolites control physiology and disease. Nat. Commun..

[43] Lee J.H., Oh S.J., Yun J., Shin O.S. (2021). Nonstructural Protein NS1 of Influenza Virus Disrupts Mitochondrial Dynamics and Enhances Mitophagy via ULK1 and BNIP3. Viruses.

[44] Zamarin D., Garcia-Sastre A., Xiao X., Wang R., Palese P. (2005). Influenza virus PB1-F2 protein induces cell death through mitochondrial ANT3 and VDAC1. PLoS Pathog..

[45] Varga Z.T., Grant A., Manicassamy B., Palese P. (2012). Influenza virus protein PB1-F2 inhibits the induction of type I interferon by binding to MAVS and decreasing mitochondrial membrane potential. J. Virol..

[46] Yoshizumi T., Ichinohe T., Sasaki O., Otera H., Kawabata S., Mihara K., Koshiba T. (2014). Influenza A virus protein PB1-F2 translocates into mitochondria via Tom40 channels and impairs innate immunity. Nat. Commun..

[47] Palmer C.S. (2022). Innate metabolic responses against viral infections. Nat. Metab..

[48] Williams N.C., O’Neill L.A.J. (2018). A Role for the Krebs Cycle Intermediate Citrate in Metabolic Reprogramming in Innate Immunity and Inflammation. Front. Immunol..

[49] Li J., Wang Y., Deng H., Li S., Qiu H.J. (2023). Cellular metabolism hijacked by viruses for immunoevasion: Potential antiviral targets. Front. Immunol..

[50] Abu-Farha M., Thanaraj T.A., Qaddoumi M.G., Hashem A., Abubaker J., Al-Mulla F. (2020). The Role of Lipid Metabolism in COVID-19 Virus Infection and as a Drug Target. Int. J. Mol. Sci..

[51] Pei Y., Robertson E.S. (2020). The Crosstalk of Epigenetics and Metabolism in Herpesvirus Infection. Viruses.

[52] Girdhar K., Powis A., Raisingani A., Chrudinova M., Huang R., Tran T., Sevgi K., Dogus Dogru Y., Altindis E. (2021). Viruses and Metabolism: The Effects of Viral Infections and Viral Insulins on Host Metabolism. Annu. Rev. Virol..

[53] Mukherjee A., Ghosh K.K., Chakrabortty S., Gulyas B., Padmanabhan P., Ball W.B. (2024). Mitochondrial Reactive Oxygen Species in Infection and Immunity. Biomolecules.

[54] Kayesh M.E.H., Kohara M., Tsukiyama-Kohara K. (2025). Effects of oxidative stress on viral infections: An overview. npj Viruses.

[55] Kirkpatrick C.T., Wang Y., Leiva Juarez M.M., Shivshankar P., Pantaleon Garcia J., Plumer A.K., Kulkarni V.V., Ware H.H., Gulraiz F., Chavez Cavasos M.A. (2018). Inducible Lung Epithelial Resistance Requires Multisource Reactive Oxygen Species Generation To Protect against Viral Infections. mBio.

[56] Manoharan R.R., Prasad A., Pospisil P., Kzhyshkowska J. (2024). ROS signaling in innate immunity via oxidative protein modifications. Front. Immunol..

[57] Fernandez-Sesma A. (2007). The influenza virus NS1 protein: Inhibitor of innate and adaptive immunity. Infect. Disord. Drug Targets.

[58] Koshiba T. (2013). Mitochondrial-mediated antiviral immunity. Biochim. Biophys. Acta.

[59] Duan X., Liu R., Lan W., Liu S. (2025). The Essential Role of Mitochondrial Dynamics in Viral Infections. Int. J. Mol. Sci..

[60] Zhirnov O.P., Konakova T.E., Wolff T., Klenk H.D. (2002). NS1 protein of influenza A virus down-regulates apoptosis. J. Virol..

[61] McLean J.E., Datan E., Matassov D., Zakeri Z.F. (2009). Lack of Bax prevents influenza A virus-induced apoptosis and causes diminished viral replication. J. Virol..

[62] Wurzer W.J., Planz O., Ehrhardt C., Giner M., Silberzahn T., Pleschka S., Ludwig S. (2003). Caspase 3 activation is essential for efficient influenza virus propagation. EMBO J..

[63] Wang Y., Hao Q., Florence J.M., Jung B.G., Kurdowska A.K., Samten B., Idell S., Tang H. (2019). Influenza Virus Infection Induces ZBP1 Expression and Necroptosis in Mouse Lungs. Front. Cell Infect. Microbiol..

[64] Goellner S., Enkavi G., Prasad V., Denolly S., Eu S., Mizzon G., Witte L., Kulig W., Uckeley Z.M., Lavacca T.M. (2023). Zika virus prM protein contains cholesterol binding motifs required for virus entry and assembly. Nat. Commun..

[65] Yuan S., Chu H., Chan J.F., Ye Z.W., Wen L., Yan B., Lai P.M., Tee K.M., Huang J., Chen D. (2019). SREBP-dependent lipidomic reprogramming as a broad-spectrum antiviral target. Nat. Commun..

[66] Sun X., Whittaker G.R. (2003). Role for influenza virus envelope cholesterol in virus entry and infection. J. Virol..

[67] Rossman J.S., Lamb R.A. (2011). Influenza virus assembly and budding. Virology.

[68] Heaton N.S., Randall G. (2011). Multifaceted roles for lipids in viral infection. Trends Microbiol..

[69] Miyanari Y., Atsuzawa K., Usuda N., Watashi K., Hishiki T., Zayas M., Bartenschlager R., Wakita T., Hijikata M., Shimotohno K. (2007). The lipid droplet is an important organelle for hepatitis C virus production. Nat. Cell Biol..

[70] Madsen J.J., Rossman J.S. (2023). Cholesterol and M2 Rendezvous in Budding and Scission of Influenza A Virus. Subcell. Biochem..

[71] Bao D., Lu C., Ma T., Xu G., Mao Y., Xin L., Niu S., Wu Z., Li X., Teng Q. (2022). Hydrophobic Residues at the Intracellular Domain of the M2 Protein Play an Important Role in Budding and Membrane Integrity of Influenza Virus. J. Virol..

[72] Kolokouris D., Kalenderoglou I.E., Duncan A.L., Corey R.A., Sansom M.S.P., Kolocouris A. (2025). The Role of Cholesterol in M2 Clustering and Viral Budding Explained. J. Chem. Theory Comput..

[73] Li X., Li L., Tian J., Su R., Sun J., Li Y., Wang L., Zhou H., Sha S., Xiao J. (2025). SREBP2-dependent lipid droplet formation enhances viral replication and deteriorates lung injury in mice following IAV infection. Emerg. Microbes Infect..

[74] Chandrasekaran P., Weiskirchen R. (2024). The Role of SCAP/SREBP as Central Regulators of Lipid Metabolism in Hepatic Steatosis. Int. J. Mol. Sci..

[75] Hendrix S., Kingma J., Ottenhoff R., Valiloo M., Svecla M., Zijlstra L.F., Sachdev V., Kovac K., Levels J.H.M., Jongejan A. (2023). Hepatic SREBP signaling requires SPRING to govern systemic lipid metabolism in mice and humans. Nat. Commun..

[76] Hale B.G., Jackson D., Chen Y.H., Lamb R.A., Randall R.E. (2006). Influenza A virus NS1 protein binds p85beta and activates phosphatidylinositol-3-kinase signaling. Proc. Natl. Acad. Sci. USA.

[77] Kang K., Reilly S.M., Karabacak V., Gangl M.R., Fitzgerald K., Hatano B., Lee C.H. (2008). Adipocyte-derived Th2 cytokines and myeloid PPARdelta regulate macrophage polarization and insulin sensitivity. Cell Metab..

[78] Cai D., Yuan M., Frantz D.F., Melendez P.A., Hansen L., Lee J., Shoelson S.E. (2005). Local and systemic insulin resistance resulting from hepatic activation of IKK-beta and NF-kappaB. Nat. Med..

[79] van Meer G., Voelker D.R., Feigenson G.W. (2008). Membrane lipids: Where they are and how they behave. Nat. Rev. Mol. Cell Biol..

[80] Harayama T., Riezman H. (2018). Understanding the diversity of membrane lipid composition. Nat. Rev. Mol. Cell Biol..

[81] Mazzon M., Mercer J. (2014). Lipid interactions during virus entry and infection. Cell Microbiol..

[82] Barman S., Nayak D.P. (2007). Lipid raft disruption by cholesterol depletion enhances influenza A virus budding from MDCK cells. J. Virol..

[83] Kawaguchi A., Hirohama M., Harada Y., Osari S., Nagata K. (2015). Influenza Virus Induces Cholesterol-Enriched Endocytic Recycling Compartments for Budozone Formation via Cell Cycle-Independent Centrosome Maturation. PLoS Pathog..

[84] Vandermeer M.L., Thomas A.R., Kamimoto L., Reingold A., Gershman K., Meek J., Farley M.M., Ryan P., Lynfield R., Baumbach J. (2012). Association between use of statins and mortality among patients hospitalized with laboratory-confirmed influenza virus infections: A multistate study. J. Infect. Dis..

[85] Paul D., Bartenschlager R. (2015). Flaviviridae Replication Organelles: Oh, What a Tangled Web We Weave. Annu. Rev. Virol..

[86] Bosch M., Sweet M.J., Parton R.G., Pol A. (2021). Lipid droplets and the host-pathogen dynamic: FATal attraction?. J. Cell Biol..

[87] Bosch M., Sanchez-Alvarez M., Fajardo A., Kapetanovic R., Steiner B., Dutra F., Moreira L., Lopez J.A., Campo R., Mari M. (2020). Mammalian lipid droplets are innate immune hubs integrating cell metabolism and host defense. Science.

[88] Zhou Y., Pu J., Wu Y. (2021). The Role of Lipid Metabolism in Influenza A Virus Infection. Pathogens.

[89] Romero-Brey I., Bartenschlager R. (2014). Membranous replication factories induced by plus-strand RNA viruses. Viruses.

[90] Halabitska I., Petakh P., Lushchak O., Kamyshna I., Oksenych V., Kamyshnyi O. (2024). Metformin in Antiviral Therapy: Evidence and Perspectives. Viruses.

[91] Lee H.S., Noh J.Y., Song J.Y., Cheong H.J., Kim W.J. (2023). Metformin reduces the risk of developing influenza A virus related cardiovascular disease. Heliyon.

[92] Bramante C.T., Beckman K.B., Mehta T., Karger A.B., Odde D.J., Tignanelli C.J., Buse J.B., Johnson D.M., Watson R.H.B., Daniel J.J. (2024). Favorable Antiviral Effect of Metformin on SARS-CoV-2 Viral Load in a Randomized, Placebo-Controlled Clinical Trial of COVID-19. Clin. Infect. Dis..

[93] Yang M., Vousden K.H. (2016). Serine and one-carbon metabolism in cancer. Nat. Rev. Cancer.

[94] DeBerardinis R.J., Cheng T. (2010). Q’s next: The diverse functions of glutamine in metabolism, cell biology and cancer. Oncogene.

[95] Altman B.J., Stine Z.E., Dang C.V. (2016). From Krebs to clinic: Glutamine metabolism to cancer therapy. Nat. Rev. Cancer.

[96] Lu S.C. (2013). Glutathione synthesis. Biochim. Biophys. Acta.

[97] Forman H.J., Zhang H., Rinna A. (2009). Glutathione: Overview of its protective roles, measurement, and biosynthesis. Mol. Asp. Med..

[98] Hong K.S., Pagan K., Whalen W., Harris R., Yang J., Stout-Delgado H., Cho S.J. (2022). The Role of Glutathione Reductase in Influenza Infection. Am. J. Respir. Cell Mol. Biol..

[99] Wise D.R., DeBerardinis R.J., Mancuso A., Sayed N., Zhang X.Y., Pfeiffer H.K., Nissim I., Daikhin E., Yudkoff M., McMahon S.B. (2008). Myc regulates a transcriptional program that stimulates mitochondrial glutaminolysis and leads to glutamine addiction. Proc. Natl. Acad. Sci. USA.

[100] Meiser J., Vazquez A. (2016). Give it or take it: The flux of one-carbon in cancer cells. FEBS J..

[101] Locasale J.W. (2013). Serine, glycine and one-carbon units: Cancer metabolism in full circle. Nat. Rev. Cancer.

[102] Pham V.N., Bruemmer K.J., Toh J.D.W., Ge E.J., Tenney L., Ward C.C., Dingler F.A., Millington C.L., Garcia-Prieto C.A., Pulos-Holmes M.C. (2023). Formaldehyde regulates S-adenosylmethionine biosynthesis and one-carbon metabolism. Science.

[103] Binkowski J., Taryma-Lesniak O., Luczkowska K., Niedzwiedz A., Lechowicz K., Strapagiel D., Jarczak J., Davalos V., Pujol A., Esteller M. (2022). Epigenetic activation of antiviral sensors and effectors of interferon response pathways during SARS-CoV-2 infection. Biomed. Pharmacother..

[104] Green R., Ireton R.C., Gale M. (2018). Interferon-stimulated genes: New platforms and computational approaches. Mamm. Genome.

[105] Marazzi I., Ho J.S., Kim J., Manicassamy B., Dewell S., Albrecht R.A., Seibert C.W., Schaefer U., Jeffrey K.L., Prinjha R.K. (2012). Suppression of the antiviral response by an influenza histone mimic. Nature.

[106] Nakaya M., Xiao Y., Zhou X., Chang J.H., Chang M., Cheng X., Blonska M., Lin X., Sun S.C. (2014). Inflammatory T cell responses rely on amino acid transporter ASCT2 facilitation of glutamine uptake and mTORC1 kinase activation. Immunity.

[107] Carr E.L., Kelman A., Wu G.S., Gopaul R., Senkevitch E., Aghvanyan A., Turay A.M., Frauwirth K.A. (2010). Glutamine uptake and metabolism are coordinately regulated by ERK/MAPK during T lymphocyte activation. J. Immunol..

[108] Ma E.H., Bantug G., Griss T., Condotta S., Johnson R.M., Samborska B., Mainolfi N., Suri V., Guak H., Balmer M.L. (2017). Serine Is an Essential Metabolite for Effector T Cell Expansion. Cell Metab..

[109] Wang R., Dillon C.P., Shi L.Z., Milasta S., Carter R., Finkelstein D., McCormick L.L., Fitzgerald P., Chi H., Munger J. (2011). The transcription factor Myc controls metabolic reprogramming upon T lymphocyte activation. Immunity.

[110] Luan H. (2025). Cell-Autonomous and Non-Cell-Autonomous Antiviral Immunity via siRNA-Directed RNAi in Drosophila melanogaster. Immune Discov..

[111] Tannahill G.M., Curtis A.M., Adamik J., Palsson-McDermott E.M., McGettrick A.F., Goel G., Frezza C., Bernard N.J., Kelly B., Foley N.H. (2013). Succinate is an inflammatory signal that induces IL-1beta through HIF-1alpha. Nature.

[112] York A.G., Williams K.J., Argus J.P., Zhou Q.D., Brar G., Vergnes L., Gray E.E., Zhen A., Wu N.C., Yamada D.H. (2015). Limiting Cholesterol Biosynthetic Flux Spontaneously Engages Type I IFN Signaling. Cell.

[113] Lu S.C., Mato J.M. (2012). S-adenosylmethionine in liver health, injury, and cancer. Physiol. Rev..

[114] Sinclair L.V., Rolf J., Emslie E., Shi Y.B., Taylor P.M., Cantrell D.A. (2013). Control of amino-acid transport by antigen receptors coordinates the metabolic reprogramming essential for T cell differentiation. Nat. Immunol..

[115] Llibre A., Kucuk S., Gope A., Certo M., Mauro C. (2025). Lactate: A key regulator of the immune response. Immunity.

[116] Hayden F.G., Shindo N. (2019). Influenza virus polymerase inhibitors in clinical development. Curr. Opin. Infect. Dis..

[117] Yang T. (2019). Baloxavir Marboxil: The First Cap-Dependent Endonuclease Inhibitor for the Treatment of Influenza. Ann. Pharmacother..

[118] Fukao K., Ando Y., Noshi T., Kitano M., Noda T., Kawai M., Yoshida R., Sato A., Shishido T., Naito A. (2019). Baloxavir marboxil, a novel cap-dependent endonuclease inhibitor potently suppresses influenza virus replication and represents therapeutic effects in both immunocompetent and immunocompromised mouse models. PLoS ONE.

[119] Andreev K., Jones J.C., Seiler P., Kandeil A., Turner J.C.M., Barman S., Rubrum A.M., Webby R.J., Govorkova E.A. (2024). Antiviral Susceptibility of Highly Pathogenic Avian Influenza A(H5N1) Viruses Circulating Globally in 2022–2023. J. Infect. Dis..

[120] Cheng M.L., Chien K.Y., Lai C.H., Li G.J., Lin J.F., Ho H.Y. (2020). Metabolic Reprogramming of Host Cells in Response to Enteroviral Infection. Cells.

[121] Vander Heiden M.G., Cantley L.C., Thompson C.B. (2009). Understanding the Warburg effect: The metabolic requirements of cell proliferation. Science.

[122] Chen P., Wu M., He Y., Jiang B., He M.L. (2023). Metabolic alterations upon SARS-CoV-2 infection and potential therapeutic targets against coronavirus infection. Signal Transduct. Target. Ther..

[123] Flores-Torres A.S., Rezinciuc S., Bezavada L., Shulkin B.L., Cormier S.A., Smallwood H.S. (2025). Respiratory Syncytial Virus Elicits Glycolytic Metabolism in Pediatric Upper and Lower Airways. Viruses.

[124] Chermahini F.A., Arvejeh P.M., Marincola F.M., Ahmad S., Naderian R., Pajand O., Eslami M., Hasannia M., Sanami S. (2025). Investigating how dengue virus-induced metabolic changes affect the host immune response and how to develop Immunomodulatory strategies. Virol. J..

